# Different profiles of immune reconstitution in children and adults with HIV-infection after highly active antiretroviral therapy

**DOI:** 10.1186/1471-2334-6-112

**Published:** 2006-07-13

**Authors:** Salvador Resino, Elena Seoane, Alicia Pérez, Ezequiel Ruiz-Mateos, Manuel Leal, Maria Á Muñoz-Fernández

**Affiliations:** 1 Laboratorio de Inmuno-Biología Molecular, Hospital General Universitario "Gregorio Marañón", Madrid, Spain; 2 Grupo de Estudio Hepatitis Vírica y SIDA, Hospital Universitario "Virgen del Rocío", Sevilla, Spain

## Abstract

**Background:**

Recent advances in characterizing the immune recovery of HIV-1-infected people have highlighted the importance of the thymus for peripheral T-cell diversity and function. The aim of this study was to investigate differences in immune reconstitution profiles after highly active antiretroviral therapy (HAART) between HIV-children and adults.

**Methods:**

HIV patients were grouped according to their previous clinical and immunological status: 9 HIV-Reconstituting-adults (HIV-Rec-adults) and 10 HIV-Reconstituting-children (HIV-Rec-children) on HAART with viral load (VL) ≤400 copies/ml and CD4^+ ^≥500 cells/μL at least during 6 months before the study and CD4^+ ^≤300 cells/μL anytime before. Fifteen healthy-adults and 20 healthy-children (control subjects) were used to calculate Z-score values to unify value scales between children and adults to make them comparable.

**Results:**

HIV-Rec-children had higher T-cell receptor excision circles (TREC) and lower interleukin (IL)-7 levels than HIV-Rec-adults (p < 0.05). When we analyzed Z-score values, HIV-Rec-children had higher TREC Z-score levels (p = 0.03) than HIV-Rec-adults but similar IL-7 Z-score levels. Regarding T-cell subsets, HIV-Rec-children had higher naïve CD4^+ ^(CD4^+^CD45RA ^hi+^CD27^+^), naïve CD8^+ ^(CD8^+^CD45RA ^hi+^CD27^+^), and memory CD8^+ ^(CD8^+^CD45RO^+^) cells/μl than HIV-Rec-adults, but similar memory CD4^+ ^(CD4^+^CD45RO^+^) counts. HIV-Rec-children had lower naïve CD8^+ ^Z-score values than HIV-Rec-adults (p = 0.05).

**Conclusion:**

Our data suggest that HIV-Rec-children had better thymic function than HIV-Rec-adults and this fact affects the peripheral T-cell subsets. Thus, T-cell recovery after HAART in HIV-Rec-adults could be the consequence of antigen-independent peripheral T-cell expansion while in HIV-Rec-children thymic output could play a predominant role in immune reconstitution.

## Background

Dysfunction of the immune system is evident in human immunodeficiency virus (HIV)-patients, even from the early phase of the HIV infection. The highly active antiretroviral therapy (HAART) dramatically affects the course of HIV-infection [1,2], because it induces a significant immunological improvement associated with suppression of viral load (VL) [3,4]. Most of HIV-patients on HAART are able to recover "normal" CD4^+ ^T-cell counts (>500 cells/μl), and a substantial increase in naïve CD4^+ ^and recent thymic emigrants expressing T-cell receptor excision circles (TREC) has been reported [5,6].

Immune recovery after HAART has been explained by either peripheral expansion of pre-existent naïve T cells, by recovery of new T cells derived from thymic production or by reversion from memory (CD45RO^+^) to naïve (CD45RA ^hi+^CD27^+^) phenotype of peripheral T cells [7]. IL-7 is very important in CD4+ recovery because it is implicated in thymopoiesis, peripheral T-cell expansion and survival of T cells. [8]. We carried out a cross-sectional study on HIV-patients (adults and children), who had recovered normal CD4^+ ^T-cell counts after HAART, grouped according to their previous clinical and immunological status to assess the different profiles of immune reconstitution.

## Methods

### Study population

We carried out a cross-sectional study on HIV-patients (adults and children) grouped according to their previous clinical and immunological status. HIV-children were recruited in the Immuno-pediatric Unit (Hospital General Universitario "Gregorio Marañón", Madrid, Spain), and all children had been infected by vertical transmission. HIV-adults were recruited in Internal Medicine Unit (Hospital Universitario "Virgen del Rocio", Seville, Spain) with the following risk groups: 6 Intravenous drug users, 2 Heterosexuals, 1 Homosexual.

HIV patients, adults and children, were selected based on the following criteria: a) CD4^+^/μL greater than 500 cells/μL; b) HIV-RNA levels below detectable limits (VL ≤400 copies/ml) at least during 6 months before the study; c) prior diagnosis of AIDS but currently asymptomatic; d) on HAART at least during 6 months before the study. Also, healthy individuals (Healthy-adults and Healthy-children) were included in the study.

This study was conducted according to the declaration of Helsinki and approved by the Ethical Committees of all hospitals involved. Written informed consent was obtained from HIV-adults and the parents/guardians of children.

Drugs were prescribed by attending physicians according to Centers for Disease Control and Prevention (CDC) guidelines [9]. Clinical classification was based on the 1994 revised guidelines of the CDC [10]. The analyses of phenotypic subsets, TRECs and IL-7 quantification were carried out in a central laboratory (Laboratorio de Immuno-Biología Molecular, Hospital General Universitario "Gregorio Marañón") using standard procedures.

### Quantification of HIV-1 RNA and IL-7 in plasma

Blood samples were collected in EDTA tubes, separated within 4 h and the plasma was stored at -70°C. Plasma VL was quantified by Amplicor standard assay (Amplicor monitor, Roche Diagnostic Systems, Brandenburg, NJ, USA). Quantification of IL-7 levels was performed in duplicate using a ELISA assay kit (Quantikine HS IL-7 kit; R&D Systems, Abingdon, UK) [5].

### Quantification of lymphocyte subsets

Lymphocyte subsets were analyzed by four-color multiparametric flow cytometry. First, the blood was lysed with ImmunoPrep™ Reagent System (Beckman-Coulter, Inc; Fullerton, CA, USA) in a Coulter^® ^TQ-Prep™ Workstation (Coulter Corporation, Miami, FL, USA). Then, the lymphocyte acquisition was carried out in a COULTER^® ^EPICS^® ^XL™ Flow Cytometer from Beckman-Coulter (Coulter Corporation) using the EXPO™32 ADC Software (Coulter Corporation) acquisition program immediately after cell staining [11]. Data were analyzed using the EXPO™32 ADC Software (Coulter Corporation) program. CYTO-COMP^® ^cell kit, CYTO-COMP^® ^reagent kit, and IMMUNOTROL^® ^were routinely used as quality controls. The monoclonal antibodies used for the analyses of lymphocyte subsets were conjugated with Fluorescein-isothyocyanate (FITC) (anti-CD45, anti-CD45RA), Phycoerytrin (PE) (anti-CD27, anti-CD4), Phycoerytrin-Texas Red^®^-x (ECD) (anti-CD8), and R-Phycoerytrin-cyanin 5.1 (PC5) (anti-CD3, anti-CD4, anti-CD8). Anti-CD27-FITC, anti-CD4-PC5, and anti-CD8-PC5 were obtained from Immunotech (Marseille, France), and anti-CD45RA-PE from Southern Biotechnology Associates (Birmingham, AL, USA). The rest of the monoclonal antibodies were obtained from Cyto-Stat^® ^Tetra-chrome™ (Coulter Corporation).

### Quantification of TCR rearrangement excision circles

Thymic function was studied by quantifying the production of TRECs in peripheral blood mononuclear cells (PBMC) by real-time quantitative PCR in a LightCycler system (Roche Molecular Biochemicals, Mannheim, Germany) [5]. Samples were analyzed in triplicate and they never varied more than 10%. A β-globin control PCR was performed to verify the presence of the same content of DNA in all samples.

### Statistical analysis

The immunological values of HIV-adults and HIV-children (Xi) were transformed to Z-scores (*Z *- *score *= (Xi-X¯)σ
 MathType@MTEF@5@5@+=feaafiart1ev1aaatCvAUfKttLearuWrP9MDH5MBPbIqV92AaeXatLxBI9gBaebbnrfifHhDYfgasaacH8akY=wiFfYdH8Gipec8Eeeu0xXdbba9frFj0=OqFfea0dXdd9vqai=hGuQ8kuc9pgc9s8qqaq=dirpe0xb9q8qiLsFr0=vr0=vr0dc8meaabaqaciaacaGaaeqabaqabeGadaaakeaadaWcaaqaamaabmaabaacbaGae8hwaGLae8xAaKMae8xla0Yaa0aaaeaacqWFybawaaaacaGLOaGaayzkaaaabaGaeq4Wdmhaaaaa@34C2@) using the mean (X¯
 MathType@MTEF@5@5@+=feaafiart1ev1aaatCvAUfKttLearuWrP9MDH5MBPbIqV92AaeXatLxBI9gBaebbnrfifHhDYfgasaacH8akY=wiFfYdH8Gipec8Eeeu0xXdbba9frFj0=OqFfea0dXdd9vqai=hGuQ8kuc9pgc9s8qqaq=dirpe0xb9q8qiLsFr0=vr0=vr0dc8meaabaqaciaacaGaaeqabaqabeGadaaakeaadaqdaaqaaGqaaiab=Hfaybaaaaa@2DFB@) and standard deviation (σ) of Healthy-adults and Healthy-children respectively to unify value scales between children and adults to make them comparable. We calculated the differences between groups using the Mann-Whitney test.

## Results

### Characteristics of HIV-1-infected patients

The HIV-patients were 9 HIV-Rec-adults (median (min; max): 40.8 (34.6; 43.7) years) and 10 HIV-Rec-children (median (min; max): 9.5 (7.1; 16.8) years) on HAART who had viral load (VL) ≤400 copies/ml and CD4+ ≥500 cells/μL at least 6 months prior to the study. Moreover, all patients had CD4+ ≤300 cells/μL and a diagnosis of AIDS at some point. These patients were compared with 15 Healthy-adults (median (min; max): 28.2 (26.3; 36.5) years) and 20 Healthy-children (median (min; max): 11.4 (6.3; 17.6) years). At the time of the study, HIV-Rec-adults and HIV-Rec-children had VL ≤400 copies/ml.

### Markers of immune reconstitution

HIV-Rec-children had lower CD4^+ ^counts (absolute and percentage) and higher %CD8^+ ^than Healthy-control, but HIV-Rec-adults had no statistical differences in CD4^+ ^absolute counts (Table 1). HIV-Rec-children did not have higher CD4^+ ^absolute counts than HIV-Rec-adults (p = 0.072). However, when we analyzed the Z-score values, startlingly, we found that HIV-Rec-children had lower CD4^+^/μl Z-score values than HIV-Rec-adults (Table 2).

**Table 1:** Summary of immunological parameters of HIV patients and HIV-seronegative individuals for the cross-sectional study.

	**HIV-Rec-adults**	**Healthy-adults**	^**(*a*)**^***p***	**HIV-Rec-children**	**Healthy-children**	^**(*b*)**^***p***	^**(*c*)**^***p***
***No. of subjects***	9	15		10	20		
***T cells***							
CD4^+ ^(%)	33.7 (25.9; 42.3)	42 (29.3; 56.6)	0.035	30.5 (18.1; 39.98)	42.3 (29.5; 55.4)	0.001	0.193
CD8^+ ^(%)	36.2 (25.7; 53.5)	22.7 (16.1; 32.3)	<0.001	38.6 (24.1; 63.9)	22.3 (13.6; 28.1)	<0.001	1
CD4^+^/μl	675 (563; 922)	864 (524; 1265)	0.815	837.5 (631; 1111)	1288 (588; 2804)	0.016	0.072
CD8^+^/μl	881 (632; 1765)	492 (196; 878)	<0.001	1038.5 (444; 2726)	673 (304; 1641)	0.091	0.620
***Thymic function***							
TRECs/10^5 ^PBMC	25 (10; 106)	247 (10; 865)	0.006	10186 (1430; 18026)	16097 (5944; 43620)	0.082	<0.001
IL-7 (pg/ml)	5.5 (2.9; 11.2)	4.3 (2.4; 7.5)	0.015	2.2 (0.8; 7.14)	0.6 (0.3; 1.8)	0.001	0.002

Values are expressed as median (minimum; maximum). TREC: TCR rearrangement excision circles. Differences were calculated by U-test of Mann-Whitney.

(a): p-values between HIV-Rec-adults and Healthy-adults.

(b): p-values between HIV-Rec-children and Healthy-children.

(c): p-values between HIV-Rec-adults and HIV-Rec-children.

**Table 2:** Summary of Z-scores of immunological markers in HIV patients for the cross-sectional study.

	**HIV-Rec-adults**	**HIV-Rec-children**	***p***
***T cells***			
CD4^+ ^(%)	-0.83 (-2.29; -0.01)	-1.56 (-3.32; -0.24)	0.079
CD8^+ ^(%)	2.03 (0.53; 5.62)	3.99 (0.38; 10.28)	0.497
CD4^+^/μl	-0.05 (-0.61; 0.43)	-0.88 (-1.2; -0.47)	**<*0.001***
CD8^+^/μl	2.12 (0.81; 7.54)	0.75 (-0.93; 5.5)	*0.053*
***Thymic function***			
TRECs/10^5 ^PBMC	-1.10 (-1.15; -0.83)	-0.74 (-1.43; -0.12)	***0.036***
IL-7 (pg/ml)	0.73 (-1.05; 4.62)	2.71 (-0.06; 12.8)	0.356
***T-cell subsets (%)***			
CD4^+^CD45RA^hi+^CD27^+^	-0.39 (-2.07; 0.7)	-0.61 (-3.17; 0.48)	0.400
CD8^+^CD45RA^hi+^CD27^+^	-1.36 (-2.52; -0.25)	-2.52 (-4.63; 0.02)	*0.053*
CD4^+^CD45RO^+^	0.95 (-1.1; 3.92)	0.06 (-2.08; 5.55)	0.243
CD8^+^CD45RO^+^	1.48 (-0.38; 6.42)	2.43 (-0.28; 5.89)	0.780
***T-cell subsets/μl***			
CD4^+^CD45RA^hi+^CD27^+^	-0.34 (-1.42; 0.62)	-0.89 (-1.23; -0.39)	0.243
CD8^+^CD45RA^hi+^CD27^+^	-0.45 (-0.9; 1.38)	-0.49 (-1.09; 0.92)	0.842
CD4^+^CD45RO^+^	-0.03 (-0.64; 0.52)	-0.54 (-1.1; 0.97)	0.095
CD8^+^CD45RO^+^	2.58 (0.61; 12.8)	1.01 (-0.5; 10.39)	0.315

Values are expressed as median (minimum; maximum). Differences were calculated by U-test of Mann-Whitney. p: p-values between HIV-Rec-adults and HIV-Rec-children.

Interestingly, HIV-Rec-adults had lower TREC values and higher IL-7 levels than healthy-adults, but HIV-Rec-children had similar TREC values and higher IL-7 levels than healthy-children (Table 1). Moreover, we observed that HIV-Rec-children had higher TREC and lower IL-7 levels than HIV-Rec-adults (Table 1). When we compared the Z-score values of HIV-Rec-adults and HIV-Rec-children, we found that HIV-Rec-children had similar IL-7 and higher TREC Z-score values than HIV-Rec-adults (Table 2).

HIV-patients had similar naïve %CD4^+ ^and lower naïve %CD8^+ ^than Healthy-controls (Table 3). However, when we analyzed the absolute counts, we found that HIV-Rec-children had lower naïve CD4^+ ^than Healthy-control but similar naïve CD8+. Moreover, HIV-Rec-children had higher naïve CD4^+ ^percentages and absolute counts, and higher naïve CD8^+ ^absolute counts than HIV-Rec-adults, but similar naïve CD8^+^percentages (Table 3).

**Table 3:** Summary of T-cell subsets of HIV patients and HIV-seronegative individuals for the cross-sectional study.

	**HIV-Rec-adults**	**Healthy-adults**	^**(*a*)**^***p***	**HIV-Rec-children**	**Healthy-children**	^**(*b*)**^***p***	^**(*c*)**^***p***
***T-cell subsets (%)***							
CD4^+^CD45RA^hi+^CD27^+^	36.8 (15.57; 51.86)	45.2 (16.5; 61.1)	0.263	56.4 (30.5; 67.4)	61.9 (45.9; 77.7)	0.080	0.009
CD8^+^CD45RA^hi+^CD27^+^	26.61 (9.82; 62.3)	41.5 (27.5; 76.8)	0.001	38.1 (15.3; 65.8)	68.9 (44; 82.7)	0.001	0.107
CD4^+^CD45RO^+^	51.2 (27.8; 85)	40.4 (21; 66.7)	0.048	41.2 (21; 92.5)	39.2 (24.8; 55.5)	0.683	0.243
CD8^+^CD45RO^+^	27.4 (11.7; 69.4)	11.3 (3.4; 32.5)	0.007	52.1 (30; 80.3)	30.2 (20.4; 48.6)	0.002	0.133
***T-cell subsets/μl***							
CD4^+^CD45RA^hi+^CD27^+^	296.8 (79.1; 493.5)	279 (172; 868.3)	0.263	445 (264; 716.6)	743.4 (289; 2062)	0.004	0.005
CD8^+^CD45RA^hi+^CD27^+^	172.6 (96.8; 519.5)	164.1 (94; 524.4)	0.861	390 (225; 777.4)	463 (156; 1005)	0.495	0.002
CD4^+^CD45RO^+^	351.8 (238.5; 453.6)	298.5 (171; 833.8)	0.682	374.8 (182.8; 894.8)	515.4 (151; 1368.6)	0.203	0.780
CD8^+^CD45RO^+^	184.2 (96.4; 640.2)	64.3 (18; 162.7)	<0.001	413.5 (153.; 2019.1)	183.7 (67; 691.6)	0.006	0.043

Values are expressed as median (minimum; maximum). Differences were calculated by U-test of Mann-Whitney.

(a): p-values between HIV-Rec-adults and Healthy-adults.

(b): p-values between HIV-Rec-children and Healthy-children.

(c): p-values between HIV-Rec-adults and HIV-Rec-children.

Regarding memory subsets, overall, HIV-patients had similar memory CD4^+ ^counts as Healthy-control; but when we analyzed memory CD8^+^, we found that HIV-patients had higher counts than Healthy-control (Table 3). Furthermore, HIV-Rec-children had similar memory CD4^+ ^percentage and absolute counts, similar memory CD8^+ ^percentage and higher memory CD8^+ ^absolute counts than HIV-Rec-adults (Table 3). Moreover, HIV-Rec-children had lower naïve CD8^+ ^Z-score values than HIV-Rec-adults (p = 0.055).

## Discussion

In humans, it is well established that HIV-1 infection adversely affects the thymus in both children and adults [7,12]. However, a recent study showed that long term survivors of paediatric HIV infection appear to have retained or recovered thymic volume and thymic activity approximating uninfected youths [13]. Yet the consequences of this thymic inhibition are worse in children, which has been proposed as one cause of the rapid progression of the disease [14-16]. Early ART in HIV-infected children has proven to induce an early immune reconstitution that correlates with the increase in thymus volume [17]. ART has also proven to be effective in recovering appropriate CD4^+ ^levels in HIV-infected patients with low CD4^+ ^counts [5,18-21], possibly through the production of new T cells in the thymus. This is particularly relevant for children, who have a smaller T-cell pool and thus rely more heavily upon thymic output for the generation of immune-competent memory T cells.

In this study, we observed that HIV-infected patients have lower thymic production than healthy controls. This inhibitory effect on the thymus may be due to the ability of HIV to infect and destroy thymocytes [22]. This would decrease the thymic production of new T cells and hinder both the appropriate maturation of the child's immune system and the reconstitution of cells lost due to the infection. When the thymic function of these HIV+ children on ART is analysed by the assessment of TRECs, it can be seen that the decrease in the viral load leads to a marked rise in thymic function [23,24], and it correlates well with the recovery of CD4^+ ^T-cell population [5,6,24-26].

In this study, we analyzed, as a whole, different aspects of immunity in HIV-1 infected patients on HAART with a previous moderate to severe immunosuppression state and who had been able to recover "normal" CD4^+ ^T-cell counts (>500 cells/μl). Interestingly, HIV-Rec-adults had lower TREC Z-score levels than HIV-Rec-children. Therefore, our results contribute new findings and extend the previous data indicating that T-cell reconstitution in HIV-adults is mainly thymus-independent [7]. However, HIV-Rec-adults had detectable TREC levels, although lower than healthy-adults, indicating their thymus was somewhat functional [11,27]. In addition, HIV-adults had naïve CD4^+ ^and CD8^+ ^counts too high to be explained by the low TREC values observed in peripheral blood. The HIV-adults could still have a defective immune system despite normal recovery of CD4^+ ^counts, although we cannot determine this since we do not have data on their TCR repertoire. Furthermore, HIV-children had a mainly thymus-dependent immune reconstitution [5,28]. Thymic function of HIV-Rec-children had a greater capacity for recovery during severe HIV-infection, and they seemed to be less affected than the HIV-Rec-adults when TREC Z-score values were compared.

Interestingly, HIV-infected patients have abnormally high plasma levels of IL-7 associated with low counts of CD4^+ ^[29]. This suggests that low CD4^+ ^counts should stimulate peripheral dendritic cells and lymphatic ganglia to produce IL-7, in an attempt to activate the mechanisms that allow cell repopulation [29]. By contrast, other studies have suggested that plasma IL-7 levels may simply reflect the dynamics of binding secreted IL-7 to T cells: the fewer circulating T cells, the greater amount of free IL-7 [30]. However, these high levels of IL-7 are ineffective in adults since no increase in thymic production or recuperation of the CD4^+ ^T-cell repertoire has been observed, possibly due to the limited ability of the adult thymus to produce new T cells.

Thus, better thymic function in HIV-children may allow for the maintaining of a high output of naïve T cells resulting in lower IL-7 levels [31]. In contrast, the thymus of HIV-adults generated less naïve T cells, making it necessary for more IL-7 to generate T cells by antigen-independent peripheral expansion [7,27]. Therefore, the high values of IL-7 could be considered normal in HIV-adults and their immune reconstitution via stimulation T cell peripheral expansion may be more IL-7-dependent. However, a role of the thymus cannot be discarded in immune reconstitution in HIV-adults.

In our experience, high levels of IL-7 in children with lower CD4^+ ^counts lead to a dramatic increase in the thymic production of new T cells and a marked recovery of CD4^+ ^counts [5]. This increase in thymic function takes place whenever viral load levels are low enough. Then, with the recovery of CD4+ counts, IL-7 returns to basal values as a consequence of a feedback mechanism [5].

Among HIV-children, we found that the thymic function of HIV-Rec-children appears to be normal, although it is well-known that HIV-infection could produce irreversible injury to the thymus of HIV-patients with AIDS [7]. However, high IL-7 values in HIV-Rec-children could increase the thymic output, increasing TRECs values and compensating for the faulty thymic function. It is also possible that IL-7 stimulates the production of T cells by antigen-independent peripheral expansion [7]. It is unclear why HIV-patients had a deficient *de novo *generation of CD4^+ ^but not of CD8^+^. Extrathymic CD8^+ ^but not CD4^+ ^lymphopoiesis has been documented in mice, which if extrapolated to humans could partially explain those differences [32,33]. Alternatively, this finding could be explained by elevated peripheral proliferation of naïve CD4^+ ^and CD8^+ ^[34].

A limitation of the study could be that immunological recovery was dependent on sustained VL suppression and efficacy of antiretrovirals that contribute to the HAART regimen. In this study, the size of groups was very small and we could not make strata according to antiretroviral therapy and sustained VL suppression periods to check the possible effect of these two factors. Another limitation of our study was that there was a difference between the age of healthy adults of the control group and HIV-Rec-adults. However, we think this difference has a low impact on the TREC levels in adults, since all of the subjects were adults inside an age range of approximately 25–40 years, Harris et al [35] in a previous study showed that thymic abundance was not inversely correlated with age in HIV-infected adults and the variation in the 25–40 year-old range is minimal.

## Conclusion

In summary, HIV-Rec-children had better thymic function than HIV-Rec-adults which affects the composition of peripheral T-cell subsets. So, T-cell recovery after HAART in HIV-Rec-adults could be the consequence of antigen-independent peripheral expansion, while neolymphopoiesis in the thymus of HIV-Rec-children could play an important role in immune reconstitution. Therefore, HIV-patients with similar CD4^+ ^counts could have CD4^+ ^of different origins and function.

## Abbreviations

**CD4**^**+**^**CD45RA**^**hi+**^**CD27**^**+ **^Naïve CD4^+ ^T-cells

**CD4**^**+**^**CD45RO**^**+ **^Memory CD4^+ ^T-cells

**CD8**^**+**^**CD45RA**^**hi+**^**CD27**^**+ **^Naïve CD8^+ ^T-cells

**CD8**^**+**^**CD45RO**^**+ **^Memory CD8^+ ^T-cells

**CDC **Centers for Disease Control and Prevention

**HAART **Highly active antiretroviral therapy

**Healthy-adults **Healthy age-matched uninfected adults as controls.

**Healthy-children **Healthy age-matched uninfected children as controls.

**HIV-Rec-adults **HIV-adults in clinical category C and CD4^+ ^T-cell ≤300 cells/μL anytime before who responded to the treatment recovering CD4^+ ^T-cell >500 cells/μL after HAART.

**HIV-Rec-children **HIV-children in clinical category C and CD4^+ ^T-cell ≤300 cells/μL anytime before who responded to the treatment recovering CD4^+ ^T-cell >500 cells/μL after HAART.

**IL **Interleukin

**PBMC **Peripheral blood mononuclear cells

**TREC **T-cell receptor excision circles

**VL **Viral load

**Z-scores ***Z *- *score *= (Xi-X¯)σ
 MathType@MTEF@5@5@+=feaafiart1ev1aaatCvAUfKttLearuWrP9MDH5MBPbIqV92AaeXatLxBI9gBaebbnrfifHhDYfgasaacH8akY=wiFfYdH8Gipec8Eeeu0xXdbba9frFj0=OqFfea0dXdd9vqai=hGuQ8kuc9pgc9s8qqaq=dirpe0xb9q8qiLsFr0=vr0=vr0dc8meaabaqaciaacaGaaeqabaqabeGadaaakeaadaWcaaqaamaabmaabaacbaGae8hwaGLae8xAaKMae8xla0Yaa0aaaeaacqWFybawaaaacaGLOaGaayzkaaaabaGaeq4Wdmhaaaaa@34C2@ using the mean (X¯
 MathType@MTEF@5@5@+=feaafiart1ev1aaatCvAUfKttLearuWrP9MDH5MBPbIqV92AaeXatLxBI9gBaebbnrfifHhDYfgasaacH8akY=wiFfYdH8Gipec8Eeeu0xXdbba9frFj0=OqFfea0dXdd9vqai=hGuQ8kuc9pgc9s8qqaq=dirpe0xb9q8qiLsFr0=vr0=vr0dc8meaabaqaciaacaGaaeqabaqabeGadaaakeaadaqdaaqaaGqaaiab=Hfaybaaaaa@2DFB@), standard deviation (σ), and values of HIV-adults and HIV-children (Xi)

## Competing interests

**Potential conflicts of interest: **The author(s) declare that they have no competing interests.

## Authors' contributions

SR had primary responsibility for protocol development, patient screening, enrollment, outcome assessment, preliminary data analysis, and contributed to the writing of the manuscript. ES, AP, and ERM had primary responsibility of ELISA, real time PCR, and flow cytometry respectively, and contributed to the writing of the manuscript. ML was responsible for patient screening, and contributed to the writing of the manuscript. MAMF supervised the design and execution of the study, the final data analyses, and the writing of the manuscript.

## Pre-publication history

The pre-publication history for this paper can be accessed here:



## References

[B1] Palella FJJ, Delaney KM, Moorman AC, Loveless MO, Fuhrer J, Satten GA, Aschman DJ, Holmberg SD (1998). Declining morbidity and mortality among patients with advanced human immunodeficiency virus infection. HIV Outpatient Study Investigators. N Engl J Med.

[B2] Resino S, Bellón JM, Resino R, Navarro ML, Ramos JT, Mellado MJ, de Jose MI, Muñoz-Fernández MA (2004). Extensive implementation of highly active antiretroviral therapy shows great effectiveness on the survival and surrogate markers in vertically HIV-infected children.. Clin Infect Dis.

[B3] Berenguer J, Perez-Elias MJ, Bellon JM, Knobel H, Rivas-Gonzalez P, Gatell JM, Miguelez M, Hernandez-Quero J, Flores J, Soriano V, Santos I, Podzamczer D, Sala M, Camba M, Resino S (2006). Effectiveness and Safety of Abacavir, Lamivudine, and Zidovudine in Antiretroviral Therapy-Naive HIV-Infected Patients: Results From a Large Multicenter Observational Cohort. J Acquir Immune Defic Syndr.

[B4] Walker AS, Doerholt K, Sharland M, Gibb DM (2004). Response to highly active antiretroviral therapy varies with age: the UK and Ireland Collaborative HIV Paediatric Study. Aids.

[B5] Correa R, Resino S, Muñoz-Fernández MA (2003). Increased Interleukin-7 Plasma Levels are Associated with Recovery of CD4+ T Cells in HIV-infected Children. J Clin Immunol.

[B6] Franco JM, Rubio A, Martinez-Moya M, Leal M, Merchante E, Sanchez-Quijano A, Lissen E (2002). T-cell repopulation and thymic volume in HIV-1-infected adult patients after highly active antiretroviral therapy. Blood.

[B7] Haynes BF, Markert ML, Sempowski GD, Patel DD, Hale LP (2000). The role of the thymus in immune reconstitution in aging, bone marrow transplantation, and HIV-1 infection. Annu Rev Immunol.

[B8] Beq S, Delfraissy JF, Theze J (2004). Interleukin-7 (IL-7): immune function, involvement in the pathogenesis of HIV infection and therapeutic potential. Eur Cytokine Netw.

[B9] CDC (2002). Guidelines for using antiretroviral agents among HIV-infected adults and adolescents. Recommendations of the Panel on Clinical Practices for Treatment of HIV. MMWR Recomm Rep.

[B10] CDCP (1994). 1994 Revised classification system for human immunodeficiency virus infection in children less than 13 years of age.. MMWR CDC Surveill Summ.

[B11] Resino S, Rivero L, Ruiz-Mateos E, Galan I, Muñoz Franco J, Muñoz-Fernández MA, Leal M (2004). Immunity in HIV-1 infected adults with a previous state of moderate-severe immune-suppression and more than 500 CD4+ T cell after highly active antiretroviral therapy. J Clin Immunol.

[B12] McCune JM (1997). Thymic function in HIV-1 disease. Semin Immunol.

[B13] Lee JC, Boechat MI, Belzer M, Church JA, De Ville J, Nielsen K, Weston S, Geng Y, Dunaway T, Kitchen C, Krogstad PA (2006). Thymic volume, T-cell populations, and parameters of thymopoiesis in adolescent and adult survivors of HIV infection acquired in infancy. Aids.

[B14] Kourtis AP, Ibegbu C, Nahmias AJ, Lee FK, Clark WS, Sawyer MK, Nesheim S (1996). Early progression of disease in HIV-infected infants with thymus dysfunction. N Engl J Med.

[B15] Nahmias AJ, Clark WS, Kourtis AP, Lee FK, Cotsonis G, Ibegbu C, Thea D, Palumbo P, Vink P, Simonds RJ, Nesheim SR (1998). Thymic dysfunction and time of infection predict mortality in human immunodeficiency virus-infected infants. CDC Perinatal AIDS Collaborative Transmission Study Group. J Infect Dis.

[B16] Meyers A, Shah A, Cleveland RH, Cranley WR, Wood B, Sunkle S, Husak S, Cooper ER (2001). Thymic size on chest radiograph and rapid disease progression in human immunodeficiency virus 1-infected children. Pediatr Infect Dis J.

[B17] Vigano A, Vella S, Saresella M, Vanzulli A, Bricalli D, Di Fabio S, Ferrante P, Andreotti M, Pirillo M, Dally LG, Clerici M, Principi N (2000). Early immune reconstitution after potent antiretroviral therapy in HIV- infected children correlates with the increase in thymus volume. AIDS.

[B18] Resino S, Galan I, Perez A, Leon JA, Seoane E, Gurbindo D, Muñoz-Fernández MA (2004). HIV-infected children with moderate-severe immune-suppression: changes in the immune system after highly active antiretroviral therapy. Clin Exp Immunol.

[B19] Bucy RP, Hockett RD, Derdeyn CA, Saag MS, Squires K, Sillers M, Mitsuyasu RT, Kilby JM (1999). Initial increase in blood CD4(+) lymphocytes after HIV antiretroviral therapy reflects redistribution from lymphoid tissues. J Clin Invest.

[B20] Resino S, Correa R, Bellon JM, Sanchez-Ramon S, Muñoz-Fernandez MA (2002). Characterizing Immune Reconstitution after Long-Term HAART in Pediatric AIDS. AIDS Res Hum Retrovir.

[B21] Correa R, Muñoz-Fernández MA (2002). Effect of HAART on Thymic Reconstitution of CD4 T lymphocytes in vertically HIV-infected Children. AIDS.

[B22] Burke AP, Anderson D, Benson W, Turnicky R, Mannan P, Liang YH, Smialek J, Virmani R (1995). Localization of human immunodeficiency virus 1 RNA in thymic tissues from asymptomatic drug addicts. Arch Pathol Lab Med.

[B23] Resino S, Galán I, Bellón JM, Navarro ML, León JA, Muñoz-Fernández MA (2003). Characterising the immune system after long-term undetectable viral load in HIV-1-infected children. J Clin Immunol.

[B24] Correa R, Munoz-Fernandez MA (2002). Production of new T cells by thymus in children: effect of HIV infection and antiretroviral therapy. Pediatr Res.

[B25] Ruiz-Mateos E, Rubio A, Vallejo A, De la Rosa R, Sanchez-Quijano A, Lissen E, Leal M (2004). Thymic volume is associated independently with the magnitude of short- and long-term repopulation of CD4+ T cells in HIV-infected adults after highly active antiretroviral therapy (HAART). Clin Exp Immunol.

[B26] Rubio A, Martinez-Moya M, Leal M, Franco JM, Ruiz-Mateos E, Merchante E, Sanchez-Quijano A, Lissen E (2002). Changes in thymus volume in adult HIV-infected patients under HAART: correlation with the T-cell repopulation. Clin Exp Immunol.

[B27] Ruiz-Mateos E, de la Rosa R, Franco JM, Martinez-Moya M, Rubio A, Soriano N, Sanchez-Quijano A, Lissen E, Leal M (2003). Endogenous IL-7 is associated with increased thymic volume in adult HIV-infected patients under highly active antiretroviral therapy. AIDS.

[B28] Resino S, Sanchez-Ramon S, Bellon JM, Correa R, Abad ML, Munoz-Fernandez MA (2002). Immunological recovery after 3 years' antiretroviral therapy in HIV-1- infected children. AIDS.

[B29] Napolitano LA, Grant RM, Deeks SG, Schmidt D, De Rosa SC, Herzenberg LA, Herndier BG, Andersson J, McCune JM (2001). Increased production of IL-7 accompanies HIV-1-mediated T-cell depletion: implications for T-cell homeostasis. Nat Med.

[B30] Bolotin E, Annett G, Parkman R, Weinberg K (1999). Serum levels of IL-7 in bone marrow transplant recipients: relationship to clinical characteristics and lymphocyte count. Bone Marrow Transplant.

[B31] Fry TJ, Connick E, Falloon J, Lederman MM, Liewehr DJ, Spritzler J, Steinberg SM, Wood LV, Yarchoan R, Zuckerman J, Landay A, Mackall CL (2001). A potential role for interleukin-7 in T-cell homeostasis. Blood.

[B32] Storek J, Joseph A, Espino G, Dawson MA, Douek DC, Sullivan KM, Flowers ME, Martin P, Mathioudakis G, Nash RA, Storb R, Appelbaum FR, Maloney DG (2001). Immunity of patients surviving 20 to 30 years after allogeneic or syngeneic bone marrow transplantation. Blood.

[B33] Dulude G, Brochu S, Fontaine P, Baron C, Gyger M, Roy DC, Perreault C (1997). Thymic and extrathymic differentiation and expansion of T lymphocytes following bone marrow transplantation in irradiated recipients. Exp Hematol.

[B34] Hazenberg MD, Otto SA, Van Rossum AM, Scherpbier HJ, De Groot R, Kuijpers TW, Lange JM, Hamann D, De Boer RJ, Borghans JA, Miedema F (2004). Establishment of the CD4+ T cell pool in healthy and untreated HIV-1 infected children. Blood.

[B35] Harris JM, Hazenberg MD, Poulin JF, Higuera-Alhino D, Schmidt D, Gotway M, McCune JM (2005). Multiparameter evaluation of human thymic function: interpretations and caveats. Clin Immunol.

